# It's time for Canadian community early warning systems for illicit drug overdoses

**DOI:** 10.1186/1477-7517-4-10

**Published:** 2007-03-28

**Authors:** Sarah J Fielden, David C Marsh

**Affiliations:** 1 Department of Interdisciplinary Studies, Institute of Health Promotion Research, University of British Columbia, 2206 East Mall, LPC 435, 4^th ^Floor, Vancouver, BC, V6T 1Z3, Canada; 2 Vancouver Coastal Health, 200-520 West 6^th ^Ave, Vancouver, BC, V5Z 4H5, Canada

## Abstract

Although fatal and non-fatal overdoses represent a significant source of morbidity and mortality, current systems of surveillance and communication in Canada provide inadequate measurement of drug trends and lack a timely response to drug-related hazards. In order for an effective early warning system for illicit drug overdoses to become a reality, a number of elements will be required: real-time epidemiologic surveillance systems for illicit drug trends and overdoses, inter-agency networks for gathering data and disseminating alerts, and mechanisms for effectively and respectfully engaging with members of drug using communities. An overdose warning system in an urban area like Vancouver would ideally be imbedded within a system that monitors drug trends and overdoses by incorporating qualitative and quantitative information obtained from multiple sources. Valuable information may be collected and disseminated through community organizations and services associated with public health, emergency health services, law enforcement, medical laboratories, emergency departments, community-based organizations, research institutions and people with addiction themselves. The present paper outlines considerations and conceptual elements required to guide implementation of such systems in Canadian cities such as Vancouver.

## Background

Illicit drug use in Canada is responsible for significant costs – both in terms of human life and healthcare resources [1,2]. The number of injection drug users alone has been estimated at 60,000–90,000 in Canada [3] and overdoses are a major cause of death in this group [3-5]. Studies indicate that drug users commonly experience and witness drug overdoses [6-8]. However, the current drug information systems provide inadequate measurement of illicit drug trends and lack the ability to detect problems and initiate a timely response to drug-related hazards such as overdoses. The present paper outlines considerations and conceptual elements for improved systems. The proposed approach pushes beyond distal epidemiological monitoring of drug trends by emphasizing a very proximal threat to public health, overdose. In this way, overdose functions as both an important indicator within drug surveillance systems as well as a health outcome requiring timely communication, intervention and preventative strategies.

## Discussion

### The Substantial Risks and Repercussions of Drug Overdose

In addition to the tragedy of overdose fatality, non-fatal overdoses amongst people with addiction users occur frequently and have been associated with high morbidity. Direct morbidity with heroin for example can include: peripheral neuropathy, gastro-intestinal problems, temporary paralysis in limbs, chest infections, and seizures while indirect complications may include physical injury due to falls, burns, and assault [7]. Factors commonly associated with increased overdose risk are: combining heroin with other central nervous system depressants such as alcohol [9], altered tolerance such as a period of abstinence from incarceration or treatment [9], high or increased heroin purity [10], and injection as route of administration [10,11]. Additional social and environmental factors that mediate overdose risk include those contextual variables such as fear of police [12], size and quality of social networks [13], homelessness [14], public injection [15], and recent life problems such as loss, health problems, and financial difficulties[16] to name a few. Popular harm reduction education messages to reduce the risk of overdose encourage people with addiction to: taste drugs before using them, do a test-shot, tourniquette-off for injection, use drugs in groups, buy drugs from a trusted source, and avoid mixing drugs with similar effects. Evaluation of a cohort of injection drug users in Vancouver found the following factors increased risk of non-fatal overdose: cocaine and heroin injection; non-injection opiate use; binge drug use; homelessness and street injection; requiring help injecting; recent incarceration; and benzodiazepine, alcohol, and speedball use; while being treated with methadone maintenance was highly protective [17].

Population-level numbers of overdoses may fluctuate due to a variety of factors such as variations in drug market trends [18], police enforcement practices [17], and temporally according to days of the week [19]. Although empirical evidence is difficult to obtain, anecdotal reports of clusters of overdoses are sometimes attributed to drug purity, for example if drugs have been cut with noxious substances or if drugs are exceptionally potent. An overdose may also occur if one drug is mistaken for another. The infiltration of "China White" (3-methylfentanyl) leading to outbreaks of overdoses in the US during the 1980s and 1990s illustrates these possibilities [20,21]. In 1989, San Francisco experienced 50 overdoses and 3 deaths over one weekend due to fentanyl [22]. Recently in Vancouver a similar fatal overdose "spike" was reported during the summer of 2005 when powdered methadone stolen from a local pharmacy was being sold as heroin, which caused a rash of 10 deaths within a two week period [23]. Vancouver and other areas of Canada have no centralized or decentralized mechanism for quickly detecting, investigating, and addressing such an outbreak.

Instances such as these create a call for greater vigilance in terms of monitoring overdoses and communicating risk to the consumers of illicit drugs as well as those service providers who work closely with people with addiction. In order for these types of early warning systems to become a reality and prove to be effective, a number of elements will be required: real-time epidemiologic surveillance systems for illicit drug trends and overdoses, inter-agency networks for gathering qualitative reports and disseminating alerts in a timely fashion, and mechanisms for engaging with members of drug using communities. These elements will look different across Canada due to the variability of provincial and municipal organization in sectors such as law enforcement and health service delivery. In Vancouver, for example, the local health authority has taken responsibility for gathering information regarding drug overdoses from health services and other sources and for issuing alerts in the community. However, in other Canadian cities, Emergency Health Services or other agencies may wish to adopt this role. Ideally, over time governments and the various sectors could collaborate across regional and provincial borders to coordinate surveillance, harmonize information systems (e.g., overdose coding and tracking) and disseminate warnings across the country.

### Current Drug Surveillance Systems Are Not Enough

Unfortunately, data surrounding drug trends and overdose prevalence and prevention remains fragmented, incomplete, and untimely in Canada and elsewhere. The US Centre for Disease Control has defined epidemiologic surveillance as "the ongoing systematic collection, analysis, and interpretation of health data essential for planning, implementation, and evaluation of public health practice, closely integrated with the timely dissemination of these data to those who need to know" [24]. In the case of illicit drug trends, no such system currently exists in Canada. The Canadian Community Epidemiologic Network on Drug Use (CCENDU) [25] tracks drug use trends in Canada using various information sources such as cohort studies, vital statistics, Ambulance Service data, population surveys, police crime statistics, and data from the Coroners Service. While this provides valuable information regarding overall past drug trends and interventions in specific areas of the country, it seems to lack the cohesion, completeness infrastructure and ability to detect and alert people in a community to drug-related hazards in a timely and coordinated manner.

Internationally, several surveillance systems are in place and could inform the development of Canadian drug information systems. However, these have limitations with regards to providing an ongoing and timely response mechanism that would be necessary to address an outbreak of overdoses. Surveillance systems for tracking drug trends include drug monitoring systems in Australia, Europe, South Africa, and the United States [19,26-30]. These use a variety of data sources such as urine and blood specimens from adult and juvenile offenders, drug use surveys, emergency department blood and urine toxicological screening, key informant interviews, focus groups and ethnographic studies. Many combine qualitative and quantitative data to provide more complete assessment of trends and risk. Drug purity data may be ascertained through drugs seized by police or through consumers providing samples that they have purchased (e.g., the Netherland's Drug Information Monitoring System or DIMS). Some of these organizations provide weekly or monthly reports although much of the reporting is done on an annual basis.

One of the more responsive systems in terms of monitoring and notification of illicit drug reactions described in the literature is the surveillance system outlined by Indig and colleagues [32]. The authors describe the coordinated effort of 15 emergency departments that were in operation for the 2000 Sydney Olympics. An Olympic Coordination Centre was established, equipped with a 24-hour phone line and connected with various services including the police and ambulance services. Self-report data regarding conditions related to illicit drugs were sent electronically within 24 hours of presentation in the emergency departments, collated and analyzed within hours, and then sent to a committee of public health experts. This type of system could be used to detect and potentially prevent overdoses by identifying problems immediately when abnormal patterns begin to appear in hospitals and communicating information back to consumers and other relevant agencies and professionals. With the 2010 Olympics taking place in Vancouver, this strategy seems feasible during the two week duration of the games; however, the feasibility of maintaining such a sentinel surveillance system over a long period of time may be limited by issues such as operational costs.

Despite the individual limitations of these international surveillance systems in terms of feasibility, comprehensiveness, accuracy, and/or ability to provide timely information back to communities, they illustrate possibilities and pitfalls that can inform the development of Canadian drug information systems. Griffith and colleagues [31] provide a comprehensive overview of the difficulties associated with current drug information systems and early detection of new drug trends. They suggest that effective drug information systems are challenged by many factors such as sociopolitical contexts, lag-time of publications, methodological complexity, the danger of raising false alarms, and knowledge being "trapped" within agencies. These factors may also hinder a rapid public health response in communities. However, these challenges do not necessarily preclude an early warning system for overdoses. They highlight the need to be creative and use multiple strategies for monitoring drug trends and overdoses and utilizing both organizational systems and human networks to collect and disseminate information.

### Inter-agency Communication Networks are Needed

Canadian drug information systems should aim to address overdose risk as quickly as possible by using both quantitative and qualitative information from multiple sectors. This includes timely access to drug testing for information on drug quality (e.g., type and purity) and information from authorities such as the theft of pharmaceuticals (e.g., from a pharmacy break-in). Including this information in an emergency warning system could act as a type of symptomatic surveillance system similar to monitoring over the counter purchases of cold remedies to predict an outbreak of influenza before it occurs [33]. Combined qualitative and quantitative information such as numbers of overdoses, unusual symptoms, location of overdoses, suspicious drugs seized by police, drug-related ambulance calls, and clinical observations in the ERs, could be reported, collated and analyzed on a daily basis as a front-line mechanism for rapidly detecting potential problems. Collection of data need not be limited to a single source such as hospitals since pooling data from all these sources could provide a more complete picture of the potential for an overdose outbreak. Reports from police, ambulance, outreach workers, healthcare workers, non-governmental organizations, general practitioners, emergency wards, and poison control could reduce the likelihood of information gaps and facilitate timely assessment of risk and a public health response. Each source of overdose information could report events (e.g., via telephone, fax, electronic forms, etc.) to a central location where they could be compared to averages to identify a potential deviation from normal. When this information is combined with qualitative reports, experts would be able to make decisions and disseminate and alert in consultation with local service agencies and people with addiction. The Canadian Adverse Drug Reaction reporting system [34] whereby consumers, healthcare professionals, and agencies can provide quantitative and qualitative reports regarding side effects of legally approved drugs and potentially activate a course of action such as issuing consumer reports might be a useful model and an untapped resource in the development of an early warning system for overdoses.

### Community Involvement is Essential

In accordance with the goals of health promotion and public health, representatives from marginalized populations should be enabled and empowered to improve their own health. The Ottawa Charter, a seminal document in Canadian health promotion policy, states that health services should be reoriented towards promoting health and sharing power with other sectors, other disciplines, and "most importantly with people themselves" and that the community should be accepted as "the essential voice in matters of its health, living conditions and well-being" [35]. The implications for the aforementioned system to prevent overdoses include involving people with drug addiction in both the reporting mechanism for exceptionally hazardous substances circulating in their communities and by targeting them in the dissemination of warnings and alerts once a threat has been detected. Involvement of people with addiction in the planning and implementation of such systems would be consistent with the recently articulated position of the Canadian Public Health Agency ("Nothing About Us Without Us") [36]. Aside from the occasional alert that is provided to the public through a sensationalized media, these types of formalized systems have not been reported. Instead, informal systems are currently responsible for spreading the warning by word of mouth through limited social networks and agency representatives that receive the warning from their clients. Although skeptics may argue that people with addiction will only use information to seek out the offending substance and cause themselves further harm, the scant evidence available suggests that only a minority of users will look for drugs they perceive as more potent [20]. Other people with addiction will likely take precautions and this could be true even for those who try to locate the drugs in question. The better informed that people are of the characteristics of the substance and the potential risks, the better able they are to make informed choices about their drug use. Involving people with addiction in the reporting and disseminating of overdose information increases the likelihood that problems are detected quickly and that messaging will be appropriate and meet the needs of the community.

Although little is known about the information networks in the drug using community, research has suggested that users learn about drug warnings through the televised and printed media, as well as from healthcare program staff, and "on the street" [20,22]. Given that many drug overdoses are never reported to emergency health services, drug users themselves may be made aware of overdose problems before anyone else becomes alerted to them. Although most overdoses are witnessed by others, bystanders will delay or neglect to seek appropriate medical assistance for reasons such as fear of arrest [37,38]. Studies indicate that many overdoses do not involve calling the ambulance or going to the hospital. For example, a recent study using data from the Vancouver Injection Drug User Study, indicated that ambulance personnel assisted in only 54% of non-fatal overdoses and only 57% were taken to hospital [39]. In addition to creating an environment that supports and enables users to seek timely medical assistance in the case of drug overdoses, promoting help-lines for adverse illicit drug reactions and encouraging users to report problems to trusted community-based organization personnel could be valuable strategies. Community-based organizations may also serve as depositories for suspected problem drugs that could undergo testing. Such a reporting mechanism could also represent another opportunity to connect drug users to harm reduction services and much needed treatment referrals.

## Conclusion

In summary, given that both fatal and non-fatal overdoses pose a significant public health concern in Canada, implementation of accurate and timely systems for monitoring and responding to drug trends and health outcomes is warranted. A local system including an urban area like Vancouver would ideally involve real-time epidemiological surveillance of drug trends and overdoses incorporating qualitative and quantitative information obtained from institutions such as emergency health services, law enforcement, laboratories, emergency departments, community-based organizations, research institutions and people with addiction themselves. Targeted warnings could be issued to various stakeholders in health, government, and the community who could then determine appropriate responses such as a mass public health warning, enhanced dissemination of harm reduction education and material, or engaging in personal risk reduction behaviours. These types of systems would complement other Canadian strategies that have been implemented to reduce drug-related harms such as methadone maintenance therapy, supervised injection facilities, needle exchange programs, and harm reduction education programs for people with addiction, meant to promote health and safety in the drug using community.

## Competing interests

The author(s) declare that they have no competing interests.

## Authors' contributions

SF conceived of the commentary and had the role of primary author in drafting and revising the manuscript. DM contributed to the intellectual content and revision of the document. Both authors read and approved the final manuscript.
